# Validation of a HPLC/FLD Method for Quantification of Tocotrienols in Human Plasma

**DOI:** 10.1155/2015/357609

**Published:** 2015-10-28

**Authors:** Hui-Ling Che, Doryn Meam-Yee Tan, Puvaneswari Meganathan, Yee-Lin Gan, Ghazali Abdul Razak, Ju-Yen Fu

**Affiliations:** ^1^Malaysian Palm Oil Board (MPOB), No. 6, Persiaran Institusi, Bandar Baru Bangi, 43000 Kajang, Selangor Darul Ehsan, Malaysia; ^2^Department of Molecular Medicine, Faculty of Medicine, University of Malaya, 50603 Kuala Lumpur, Malaysia; ^3^International Medical University, No. 126, Jalan 19/155B, Bukit Jalil, 57000 Kuala Lumpur, Malaysia; ^4^Department of Surgery, Faculty of Medicine, University of Malaya, 50603 Kuala Lumpur, Malaysia; ^5^Department of Bioprocess Technology, Faculty of Biotechnology and Biomolecular Sciences, Universiti Putra Malaysia, 43400 Serdang, Selangor, Malaysia

## Abstract

Quantification of tocotrienols in human plasma is critical when the attention towards tocotrienols on its distinctive properties is arising. We aim to develop a simple and practical normal-phase high performance liquid chromatography method to quantify the amount of four tocotrienol homologues in human plasma. Using both the external and internal standards, tocotrienol homologues were quantified via a normal-phase high performance liquid chromatography with fluorescence detector maintained at the excitation wavelength of 295 nm and the emission wavelength of 325 nm. The four tocotrienol homologues were well separated within 30 minutes. A large interindividual variation between subjects was observed as the absorption of tocotrienols is dependent on food matrix and gut lipolysis. The accuracies of lower and upper limit of quantification ranged between 92% and 109% for intraday assays and 90% and 112% for interday assays. This method was successfully applied to quantify the total amount of four tocotrienol homologues in human plasma.

## 1. Introduction

Natural occurring vitamin E, or chemically known as tocochromanols, comprises of eight molecules having a 16-carbon chain attached to a chromanol ring. Among them, tocopherols (TP) have saturated carbon side chain whereas tocotrienols (T3) contain three* trans*double bonds in their carbon tails. Homologues of TP and T3, namely, *α*-, *β*-, *γ*-, and *δ*-homologues, are categorized based on the number and position of methyl groups on the chromanol ring [1, 2]. The unsaturated carbon side chains present in tocotrienols give rise to their unique biological functions. In addition to their antioxidant properties, T3 have been widely investigated for their anticancer and neuroprotection as well as cholesterol lowering effects [3, 4]. The distinctive properties of T3 had gained much attention in the research community in recent years, leading to the emergence of numerous human clinical trials. There is a critical need for a validated and optimized method for accurate quantification of T3 in biological fluids, especially human plasma.

In 1997, the American Oil Chemists' Society (AOCS) published an official method for quantification of vitamin E using high performance liquid chromatography (HPLC), suitable for detection in vegetable oils and fats. However, the direct application of this method on biological samples is limited due to the interference from blood proteins and the lack of sensitivity to detect small quantity. In the AOCS method, *α*-TP were used as reference as T3 standards were not available. Ng et al. [5] reported that when calibrated using *α*-TP as standard, the concentrations of vitamin E homologues appeared to be overestimated. A calibrated method using individual T3 homologues as standards is strongly recommended for accurate measurement of T3 concentrations. In addition, fluorescence detector gives higher sensitivity compared to UV detector as reported in AOCS method, enabling accurate measurement of small quantity in blood samples.

Over the years, several methods were developed for the quantification of vitamin E using HPLC. Most of these methods aim to determine the concentration of TP and T3 in food samples, including hazelnuts [6], olive oils [7], palm oils [8], and cereals [9]. A method using reverse phase-HPLC was reported by Yap et al. [10], to detect three T3 homologues as well as *α*-TP in human blood samples. Lee et al. [11] have developed a method that was able to quantify vitamins A and E and various diet-derived carotenoids in human plasma. However, the method involved two different analytical columns and detectors. On the other hand, Abuasal and his coworkers have reported a specific *γ*-T3 analysis that can be adapted in both human and rat plasma [12]. In this study, we developed and validated a normal-phase-HPLC system with well-separated chromatograms able to detect small quantity of T3 in human blood samples.

## 2. Materials and Methods

### 2.1. Chemicals and Reagents

A mixed tocotrienols-tocopherols complex 50%  oil suspension containing 11.51%  *α*-tocopherol, 12.99%  *α*-tocotrienol, 2.55%  *β*-tocotrienol, 19.48%  *γ*-tocotrienol, and 7.28%  *δ*-tocotrienol was used as an external standard. Internal standard 2,2,5,7,8-pentamethyl-6-chromanol (PMC) and sodium chloride were purchased from Sigma-Aldrich (Malaysia) Sdn. Bhd. N-Hexane, 1,4-dioxane, 2-propanol, and ethanol (*LiChrosolv* grade) were purchased from Merck Sdn. Bhd. (Malaysia). Blank human plasma was obtained from Transfusion Medicine, University Malaya Medical Centre, Malaysia.

### 2.2. Instrumentation

TP and T3 homologues were analysed using Agilent 1100 series HPLC system equipped with quaternary pump (G1311A, serial number DE91609486), fluorescence detector (G1321A, serial number DE92002049), degasser (G1322A, serial number JP73021896), autosampler (G1313A, serial number DE91611414), and* ChemStation* Software. Chromatographic separations were performed using Luna 5u Silica 100A ODS (250 × 4.60 mm·d, 5 *μ*m particle size) Hypersil column (*Phenomenex*, USA). Column was maintained at a pressure of 24 ± 1 bar and a temperature of 26°C. Samples were injected at a volume of 100 *μ*L with flow rate of 1 mL/min and a total run time of 30 minutes. Mobile phase was prepared using n-Hexane, 1,4-dioxane, and 2-propanol at 97.5 : 2.0 : 0.5%  v/v/v, degassed by sonication prior to use. Vitamin E homologues were detected using fluorescence detector at excitation wavelength 295 nm and emission wavelength 325 nm (PMT-Gain at 10).

### 2.3. Preparation of Standard Solutions

Tocotrienols-tocopherols complex 50%  oil suspension standard was prepared in a stock solution at 1000 ppm in ethanol. Working standard solution of 100 ppm was prepared by diluting stock standard solution in human plasma. Six dilutions ranging from 100, 75, 50, 10, and 5 to 1 ppm were prepared in human plasma. PMC as internal standard (IS) was prepared in mobile phase at 2000 ppm as a stock solution. Working standard solutions (10 ppm) were prepared by serial dilution in mobile phase.

### 2.4. Preparation of Human Plasma Samples

Human plasma samples were prepared according to method previously reported by Nesaretnam et al. [13] with slight modification. A volume of 0.5 mL of human plasma was added into a 12 mL glass test tube before spiking with 0.05 mL of 10 ppm IS. The mixture was vortexed for 10 seconds and centrifuged at 2500 rpm for 2 minutes at 4°C. One mL of 0.9% sodium chloride (NaCl) was added and vortexed vigorously, followed by an addition of 1 mL ethanol. The mixture was vortexed. Then, 5 mL of n-hexane was added and the mixture was shaken for one hour at 1400 rpm by using a mini shaker (IKA-VIBRAX-VXR, IKA, Germany). The mixture was centrifuged at 2500 rpm for 15 minutes with a temperature of 4°C, after which the upper organic layer was extracted and evaporated to dryness with nitrogen gas. Dried samples were reconstituted with 0.5 mL of mobile phase prior to analysis. Samples were analysed at *n* = 1 and average plasma levels of tocotrienol homologues from 3 volunteers were determined.

### 2.5. Detection Range

Calibration curves were constructed using six standard concentrations of total tocotrienols ranging from 1 to 100 ppm. Peak areas of *α*-tocotrienol, *γ*-tocotrienol, *δ*-tocotrienol, and IS were plotted against their nominal concentrations and calibration curves were constructed based on linear equation *y* = *mx* + *c*, where *y* represents the peak area, *x* represents the nominal concentration, *m* represents the slope, and *c* represents the *y*-intercept values. Linear regression was evaluated using correlation coefficient (*R* ^2^). Selectivity and specificity were determined with analysis of blank plasma. Limit of detection (LOD) was determined using lowest concentration with peak area of signal-to-noise (S/N) ratio of ≥3. Limit of quantification (LOQ) was referred to as the lowest concentration on calibration curve at which quantitative results can be reported with a high degree of confidence that produced a peak with S/N ratio of ≥10.

### 2.6. Precision and Accuracy

Precision and accuracy of the method were evaluated by analysing the lower limit of quantification (LLOQ) and upper limit of quantification (ULOQ). Three replicates of LLOQ and ULOQ were analysed within the same day for intraday (within-day) precision. Interday (between-day) precision was obtained by analysing three replicates of LLOQ and ULOQ on consecutive days. Intra- and interday precision were expressed as the percentage of relative standard deviation (%RSD). Deviation from the true value was determined by comparing the obtained concentration with nominal concentration for intraday and interday accuracy and expressed as % accuracy.

### 2.7. Supplementation of Tocotrienols

Subjects with metabolic syndrome were recruited from a Malaysian population with age category ranging from 25 to 56 years. Ethical approval was obtained from the ethical committee from the University Putra Malaysia and registered at https://clinicaltrials.gov/ (NCT01631838). Subjects were supplemented with tocotrienol (Tocovid Suprabio) 200 mg twice daily for 14 days. Blood samples were collected into K3EDTA vacutainer 4 hours after consumption of capsules on day 14. Plasma samples were collected using centrifugation at 3000 rpm for 14 minutes at 4°C. All human plasma samples were snap-frozen in liquid nitrogen and stored in −80°C until analysis. The study was conducted according to Good Clinical Practice and Declaration of Helsinki guidelines 2008.

## 3. Results and Discussion 

### 3.1. Selectivity and Specificity

**Figure 1:**
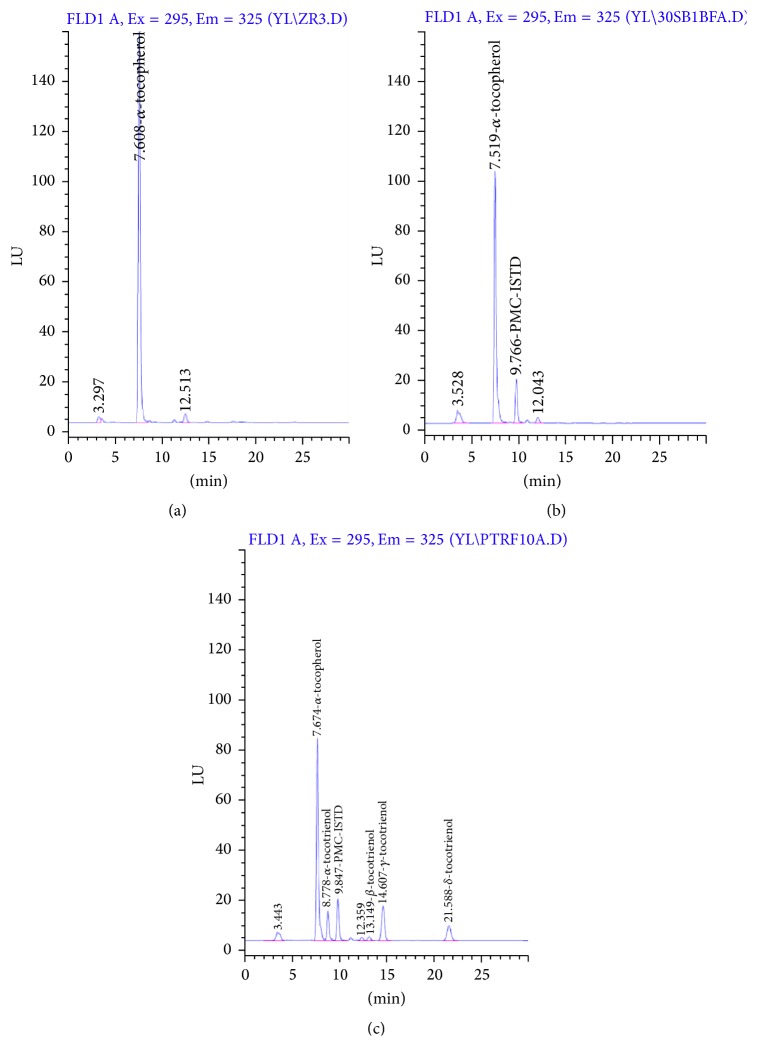
Chromatograms of (a) blank plasma, (b) plasma spiked with IS, and (c) plasma spiked with IS and vitamin E standard.

Figure 1 illustrates the chromatograms of blank plasma and plasma spiked with IS and mixed tocopherol/tocotrienol standard. From the chromatogram of blank plasma, selectivity was verified as no interference peak was observed except for endogenous *α*-TP and an unknown peak, which was detected at 7.9 and 12.5 minutes, respectively. The long elimination half-life of *α*-TP, with reported value of around 20 h, is the main reason for the presence of *α*-TP peak in blank plasma [14, 15]. This is similar to the results reported by several clinical studies [10, 16, 17]. As such, the current method is not ideal to quantify *α*-TP in plasma. Future efforts for calibration using synthetic plasma [18] or plasma stripped off endogenous compounds are warranted. Specificity of IS and T3 homologues was determined in Figures 1(b) and 1(c). Retention time of IS was observed at 9.8 minutes, in the absence of endogenous interference. The elution of T3 homologues followed the order of *α*-T3, *β*-T3, *γ*-T3, and *δ*-T3 at 8.8, 13.1, 14.6, and 21.6 minutes, respectively. Although all peaks were generally well separated, the retention time for *β*-T3 and unknown peak in the plasma were relatively close. Identification of the unknown peak is warranted in future studies. Peak resolution of *α*-TP can be improved as tailing effect was observed. According to Ng and Yuen May [19], a derivative of tocopherols, tocomonoenol, was eluted between *α*-TP and *α*-T3. The peak of tocomonoenol might be resolved with slight modification in the chromatographic separation of the current method to avoid overestimation of *α*-TP. Nevertheless, limited availability of tocomonoenol standard might hamper the identification process.

### 3.2. Detection Range

Calibration curves were constructed using six standard concentrations ranging from 1 to 100 ppm for total tocotrienols. For individual tocotrienol homologues, the detection range (0.02 ppm to 20 ppm) was summarized in Table 1. Variations in peak area for IS were relatively small (ranges between 5–8 LU) within-day and between-day. As such, calibration curves were constructed based on peak area against nominal concentrations of T3. However, it is reasonable to construct calibration curves using the peak area ratios of T3 homologues against IS in future work in order to eliminate the variations in peak area. In this method, the correlation coefficients (*R* ^2^) for all T3 homologues were higher than 0.999, which were sufficiently high to indicate a linear correlation between the peak area and T3 concentrations. On the other hand, each T3 homologue varied in their slope and *y*-intercept values (Table 1). Our results correlated with recommendations by Ng and Yuen May [19] that quantification of vitamin E homologue should be correlated with their own standards to avoid over- or underestimation. The LLOQ and ULOQ of total tocotrienols were determined at 1 ppm and 100 ppm for total tocotrienols, respectively. For individual homologues, the LLOQ and ULOQ correlate with the calibration range summarized in Table 1.  Table 2 summarizes the LOD and LOQ of T3 homologues in human plasma. Chromatogram of LOD/LOQ was presented in Supplementary Data (in Supplementary Material available online at http://dx.doi.org/10.1155/2015/357609). All T3 homologues appeared to have similar LOD and LOQ as dilutions below the LOQ did not yield S/N ratio of ≥3. The LOD and LOQ observed ranged from 0.0255 ppm in *β*-T3 to 0.1948 ppm in *γ*-T3, higher values than that reported by Yap et al. [10], Lee et al. [11], and Abuasal et al. [12]. Nevertheless, S/N ratio of *β*-T3 was relatively low compared to the other T3 homologues due to its low concentration in the tocotrienols-tocopherols complex 50% standard. Using pure standards of individual T3 homologues will significantly improve the accuracy of T3 quantification in future work.

### 3.3. Precision and Accuracy

Precision of the method was determined by quantifying the %RSD of LLOQ and ULOQ in intra- and interday analysis (Table 3). The %RSD was found to be less than 5% for both LLOQ and ULUQ at intra- and interday analysis. The variation is marginally higher than 2% as recommended by the FDA Guidance for Industry on Bioanalytical Method Validation for bioequivalence. The inconsistency in injection volume and detector might give rise to the variation, especially for low concentrations in LLOQ. The precision of this method can be improved by calibrating using area ratio of analyte peak to internal standard peak. The area ratio calibration is more reproducible and eliminates the instrumentation inconsistency [20]. Accuracy of LLOQ and ULOQ ranged between 92 and 109% for intraday assays and 90 and 112% for interday assays. The International Conference on Harmonisation of Technical Requirements for Registration of Pharmaceuticals for Human Use (ICH) guidelines suggested % accuracy within 80–120% for reliable estimation of the true values.

### 3.4. Concentration of Tocotrienols in Human Plasma Samples

**Figure 2:**
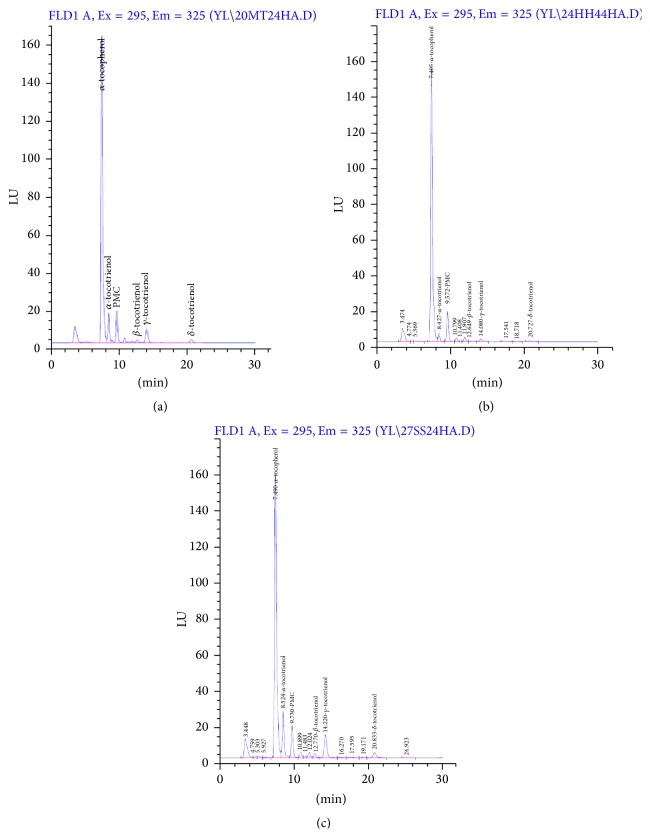
Chromatograms for human plasma of three male volunteers (a–c) after consumption of 200 mg mixed tocotrienols.

Figure 2 illustrates the chromatograms for human plasma of three male volunteers after consumption of 200 mg mixed tocotrienols. At 4 hours after supplementation, the total amount of T3 detected in plasma ranged from 0.93 ppm to 6.13 ppm. According to Yap et al. [10] and Fairus et al. [21], highest plasma concentration of T3 (*T*_max_) was reached at 4-5 hours, dependent on the different homologues. A large interindividual variation between subjects was observed. This phenomenon was previously reported by Heng et al. [22], as tocotrienols were absorbed via the lipoprotein pathway, which is dependent on food matrix and gut lipolysis [23].  Table 4 summarizes the levels of different T3 homologues detected. Alpha-T3 and *γ*-T3 appeared to be most abundant, correlating with their high amount in Tocovid Suprabio capsules (61.52 mg *α*-T3, 112.80 mg *γ*-T3, and 25.68 mg *δ*-T3). The recovery of IS was calculated at 100%, 119%, and 128%, respectively, for subjects A, B, and C. Three unidentified peaks were detected at approximately 10.8, 11.9, and 17.6 minutes. They were postulated as *β*-, *γ*-, and *δ*-tocopherols although further identification is needed with respective standards.

## 4. Conclusion

A simple, specific, and reproducible HPLC method was developed and validated for detection of tocotrienols in human plasma. The method is selective and specific for detection of *α*-, *β*-, *γ*-, and *δ*-T3 in human plasma. Validation of the method showed sufficient precision and accuracy at a concentration ranging from 0.02 to 20 ppm. Stability tests upon freeze-thaw cycles and ambient temperatures are warranted to verify the conditions of analysis and sample storage. The current method has been applied for quantification of tocotrienol levels in plasma of human volunteers consuming mixed tocotrienols. This method will be useful for clinical studies for accurate quantification of tocotrienols in biological fluids, especially human plasma.

## Supplementary Material

Chromatogram for LOD and LOQ of T3 homologues, which corresponds to Table 2 at dilution point of total T3 standards at 1ppm was provided as supplementary material.

## Figures and Tables

**Table 1:** Detection range and linear correlation of T3 homologues.

T3 homologues	Calibration range (ppm)	Slope (*m*)	*Y*-intercept (*c*)	*R* ^2^
*α*-T3	0.1299–12.9900	144.5622	5.8068	0.9994
*β*-T3	0.0255–2.5500	121.2506	1.4434	0.9995
*γ*-T3	0.1948–19.4800	150.5562	3.1899	0.9997
*δ*-T3	0.0728–7.2800	232.6348	2.0644	0.9997

**Table 2:** LOD and LOQ of T3 homologues.

T3 homologues	LOD (ppm)	LOQ (ppm)
*α*-T3	0.1299	0.1299
*β*-T3	0.0255	0.0255
*γ*-T3	0.1948	0.1948
*δ*-T3	0.0728	0.0728

**Table 3:** Precision and accuracy of LLOQ and ULOQ.

	Precision (%RSD)	Accuracy (% accuracy)
Intraday assay		
LLOQ	3.97	109.65
ULOQ	0.34	92.87
Interday assay		
LLOQ	2.00	112.81
ULOQ	2.13	90.11

**Table 4:** Average concentrations of T3 homologues detected in human plasma of 3 male volunteers.

T3 homologues	Subject A (ppm)	Subject B (ppm)	Subject C (ppm)	Mean (ppm)	SD (ppm)
*α*-T3	1.7423	0.5305	3.0948	1.7892	1.283
*β*-T3	0.3059	0.0877	0.4383	0.2773	0.177
*γ*-T3	1.2006	0.2521	2.2102	1.2210	0.979
*δ*-T3	0.2221	0.0631	0.3893	0.2248	0.163
