# Supplementary material for: Validation of a HPLC/FLD Method for Quantification of Tocotrienols in Human Plasma
Source: Int J Anal Chem. 2015 Oct 28;2015:357609. doi: 10.1155/2015/357609 (PMC4641197; doi:10.1155/2015/357609)
Supplement: Supplementary file 1 — Chromatogram for LOD and LOQ of T3 homologues, which corresponds to Table 2 at dilution point of total T3 standards at 1ppm was provided as supplementary material. [file 357609.f1.docx]

**Supplementary Data**

Figure S1: Chromatogram of LOD and LOQ of T3 homologues (corresponding to 1ppm of total T3 standard)
